# Clinical Implications of Epigenetic Dysregulation in Perinatal Hypoxic-Ischemic Brain Damage

**DOI:** 10.3389/fneur.2020.00483

**Published:** 2020-06-09

**Authors:** Martín Bustelo, Melinda Barkhuizen, Daniel L. A. van den Hove, Harry Wilhelm. M. Steinbusch, Martín A. Bruno, C. Fabián Loidl, Antonio W. Danilo Gavilanes

**Affiliations:** ^1^Department of Pediatrics, Maastricht University Medical Center (MUMC), Maastricht, Netherlands; ^2^Department of Psychiatry and Neuropsychology, School for Mental Health and Neuroscience (MHeNs), Maastricht University, Maastricht, Netherlands; ^3^Instituto de Ciencias Biomédicas, Facultad de Ciencias Médicas, Universidad Católica de Cuyo, San Juan, Argentina; ^4^Laboratorio de Neuropatología Experimental, Facultad de Medicina, Instituto de Biología Celular y Neurociencias “Prof. E. De Robertis” (IBCN), Universidad de Buenos Aires, CONICET, Buenos Aires, Argentina; ^5^Department of Psychiatry, Psychosomatics and Psychotherapy, University of Würzburg, Würzburg, Germany; ^6^Facultad de Ciencias Médicas, Instituto de Investigación e Innovación de Salud Integral, Universidad Católica de Santiago de Guayaquil, Guayaquil, Ecuador

**Keywords:** hypoxic-ischemic encephalopathy, biomarker, hypoxia, ischemia, microRNAs, histone modifications, DNA methylation

## Abstract

Placental and fetal hypoxia caused by perinatal hypoxic-ischemic events are major causes of stillbirth, neonatal morbidity, and long-term neurological sequelae among surviving neonates. Brain hypoxia and associated pathological processes such as excitotoxicity, apoptosis, necrosis, and inflammation, are associated with lasting disruptions in epigenetic control of gene expression contributing to neurological dysfunction. Recent studies have pointed to DNA (de)methylation, histone modifications, and non-coding RNAs as crucial components of hypoxic-ischemic encephalopathy (HIE). The understanding of epigenetic dysregulation in HIE is essential in the development of new clinical interventions for perinatal HIE. Here, we summarize our current understanding of epigenetic mechanisms underlying the molecular pathology of HI brain damage and its clinical implications in terms of new diagnostic, prognostic, and therapeutic tools.

## Introduction

Epigenetics is defined as heritable changes in gene expression that do not result from a change in the DNA sequence (1). Epigenetic regulation plays an essential role during development, and any insult that disrupts physiological developmental epigenetic programming is likely to have long-term consequences. An increasing number of studies link exposure to different adverse factors during early life, including both the gestational and the postnatal period to changes in the epigenome and one's individual suceptibility (2). In this regard, the brain is particularly vulnerable to alterations in the early-life microenvironment, damage induced at this stage may not be evident until the exposure to a new insult triggers it (3).

Perinatal hypoxia represents one of the most common early life insults that ultimately leads to disability or even early death (4, 5). Hypoxia can occur progressively during pregnancy in cases of fetal growth restriction due to placental abnormalities, or can occur acutely during labor and birth, causing peripartum hypoxic-ischemic encephalopathy (HIE). Fetal growth restriction is associated with stillbirths and permanent neurological disability in survivors (6). Management of fetal growth restriction depends on early detection, and timely delivery before stillbirth occurs (7). Advances in this area have focused on detecting the presence of fetal growth restriction, and the degree of hypoxia present *in utero*.

HIE has a large impact on global child health, with morbidity in 2.5/1,000 live births (4–9 million newborns affected per year worldwide) (8–10). At present, therapeutic hypothermia is the only approved therapy for newborns ≥36 weeks gestational age with moderate-to-severe HIE. Therapeutic hypothermia is thought to work by generally slowing down metabolism and thus counteracting a variety of pathological mechanisms of HIE (11). Several trials have demonstrated that hypothermia is effective in decreasing mortality and decreasing neurocognitive impairments (12, 13), still, this therapy is only partially protective, half of treated newborns still die or develop a lifelong disability, demonstrating the need for the development of other neuroprotective treatment strategies (14).

The features of acute intrapartum hypoxic-ischemia (HI) involve fetal hypoxemia, hypercapnia, and ischemia. In the brain, this leads to metabolic acidosis, cellular necrosis, and activation of apoptotic pathways. After reperfusion/reoxygenation, oxidative metabolism recovers in surviving cells, and most of the neurotoxic cascade is seemingly terminated. This first period immediately following the HI event (0–6 h) is also referred to as the “therapeutic window,” where intervention may prevent secondary damage.

Secondary energy failure (6–48 h following HI) involves potent inflammation as well as oxidative stress induced by reactive oxygen species and free radicals, and failure of mitochondrial oxidative phosphorylation due to permeabilization of the mitochondrial membranes. Ultimately, these processes lead to delayed cell death via necrotic and apoptotic pathways (15). Brain injury continues to evolve even months and years after the initial insult, in the tertiary phase, involving neural scarring and persistent inflammation (16). Traditionally epigenetic changes have been attributed to the tertiary phase, its role in the initial phases of HI induced injury is less well-characterized.

Different *in vivo* and *in vitro* models have been used to study the pathological features of HIE. Rodents are the most commonly used animals to model perinatal HI (17), with three major approaches being used. The first model makes use of submersion of the uterine horns containing the term fetal rats in saline. This model mimics a global insult to the fetus at a very low gestational age (18, 19). Considering the fact that brain development in rodents is delayed when compared to that in humans (17), other models use neonatal pups at Postnatal day (P) 3–10 (20) to model insults in the late preterm to term brain. Amongst others, neonatal pups can be subjected to a global hypoxic insult by placing them in a hypoxic chamber, an approach, however, that lacks the ischemic nature of the insult seen in the clinic (20). The most commonly used model, developed by Levine in adult rodents, and adapted by Rice and Vannucci for neonatal rodents (21, 22), induces ischemia by carotid artery occlusion (CAO) followed by exposure to systemic hypoxia (23) inducing an HI insult, here referred as the “HI” model. *In vitro* models exposing neuronal cultures to oxygen-glucose deprivation (OGD)/reoxygenation have also been used as models of HI induced injury (24).

## Epigenetic Dysregulation in Hypoxia-Ischemia

**Figure 1:**
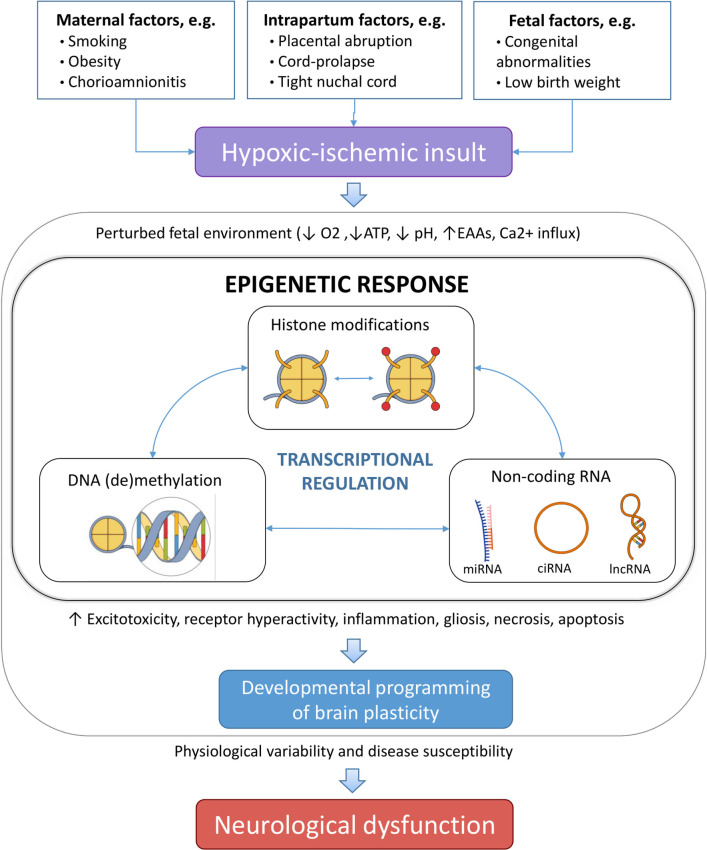
Different risk factors, which can be ante-, peri-, or postnatal can lead to perinatal hypoxia. If prolonged hypoxia results in a perturbed brain environment, leading to modulated epigenetic changes that alter the gene expression profile as an adaption of the fetus to the adverse environment. Different epigenetic mechanisms cooperatively orchestrate this process. As a result, changes in physiology of the neonatal brain can permanently affect the structure and/or functionality, and result in increased disease susceptibility in the offspring. EAAs, excitatory amino acids; miRNA, microRNA; ciRNA, circular RNA; lncRNA, long non-coding RNA.

Epigenetic processes regulate both the transcription of the DNA into mRNA and the translation of the mRNA into proteins, acting at multiple levels of control, involving e.g., DNA methylation and hydroxymethylation, chromatin remodeling, and non-coding RNA (ncRNA) regulation (25, 26). As such, DNA is wrapped around histone proteins forming chromatin, where the configuration of chromatin is controlled by post-translational modifications of the associated histone proteins as well as by direct modifications to nucleotides, which collectively form the epigenetic code that controls the transcription of DNA into mRNA (25). This epigenetic code can be modified by “writers,” i.e., enzymes which introduce post-translational modifications on the DNA and histones, “erasers,” which remove the modifications, and “readers,” i.e., specialized proteins which identify and interpret the modifications. The translation of mRNA into proteins is regulated by ncRNAs. HI-induced epigenetic alterations are preserved even in the absence of hypoxia-inducible factors (HIFs) (27, 28) suggesting they may continue to impact upon behavioral phenotypes later in life (Figure 1).

### Hypoxia-Inducible Factor-1

Hypoxia-inducible factor-1 (HIF-1) is the main effector of cellular hypoxia, being an oxygen-sensitive transcription factor. HIF-1 comprises two subunits, i.e., HIF-1α and HIF-1β (29). The stability and activity of the oxygen-regulated subunit HIF-1α are regulated by post-translational modifications that control its proteasomal degradation. In hypoxia, activation of HIFs is mediated via the inhibition of dioxygenases, such as the Jumonji-C (JmjC) domain-containing histone demethylases. As a result, HIF-1α accumulates, binds to a hypoxic response element (HRE), and leads to the transcriptional induction of several genes involved in adaptation to hypoxia, including genes involved in angiogenesis, iron metabolism, and glucose metabolism.

HIF-1 expression levels, protein stabilization, and its association with HRE are under tight epigenetic regulation, including promoter methylation and microRNA (miRNAs) control (30–32). Conversely, HIF-1 controls the expression of several epigenetic regulators. Studies have shown that DNA methylation regulates HIF-1α transcription regulating the promoter activity at HRE-152 (33) and SP1.

### DNA Methylation

DNA methylation comprises the addition of a methyl group at the carbon 5 position on the pyrimidine ring of cytosines, creating 5-methylcytosine (5-mC) (34). Cytosine methylation provides a stable epigenetic mark with long-term transcriptional effects. DNA methylation is catalyzed by DNA methyltransferases (DNMTs). These enzymes can work in different ways depending on the local chromatin microenvironment, acting as DNA methyltransferase or DNA dehydroxymethylase (35). The methylation status of the DNA is “read” by methyl CpG binding proteins (MBPs), like the methyl-CpG-binding protein 2 (MECP2) which recruits HDACs to repress transcription (36). Recent evidence indicates that during early postnatal development neuronal genomes accumulate uniquely high levels of two alternative forms of methylation, i.e., non-CpG methylation and DNA hydroxymethylation (37). DNA methylation marks can be “erased” either passively through inhibition of the DNMTs or actively by Ten-eleven translocation (TET) enzymes. TETs are 2-oxoglutarate-dependent dioxygenases that mediate DNA hydroxymethylation and their activity is oxygen-dependent (38).

### Non-coding RNAs

In humans, only 1–2% of the genome encodes proteins, while non-coding RNAs (ncRNAs) that do not produce proteins are the great majority of human transcripts (39). As such, ncRNAs, including microRNAs (miRNAs), long non-coding RNAs (lncRNAs), small nucleolar RNAs, and circular RNAs (circRNAs), have important roles in regulating gene expression playing diverse regulatory roles (40). The limited availability of human fetal and neonatal tissue has resulted in studies using animal models and *in vitro* studies as a major alternative for studying the role of ncRNAs in newborns under physiological and pathophysiological conditions.

#### miRNAs

MiRNAs are a large class of short regulatory RNAs, about 19–22 nucleotides in length, which silence gene expression by binding to the 3′-untranslated region of target genes. The majority of miRNAs are enriched in the developing brain, which signifies their role in neural development (41). Consequently, disruption of miRNAs in the perinatal period is likely to have long-term consequences (42, 43). Several miRNAs termed hypoxamirs have been shown to be induced by hypoxia, and control the cellular response to hypoxia via HIFs. These include miRNAs that regulate HIF1-α signaling (31, 44–50) and miRNAs that contain hypoxia-responsive elements that are transcribed in response to HIF1-α activation and act downstream of the HIFs (31, 47, 49, 51–53). miRNA expression is highly tissue- and disease-specific, which, together with their remarkable stability in circulation, makes them potential diagnostic biomarker candidates, still none has made it to a clinical setting (Table 1).

**Table 1:** Clinical implications of non-coding RNAs in perinatal hypoxia ischemia studies: SYMBOLS: ↑, Upregulation; ↓, Downregulation; –, Not specified.

**Observed in**		**Observed effect in HI**	**Clinical implications**	**References**
Human	Blood plasma	↑miR-210	Therapeutic target	(54)
	Placenta			(55)
			Biomarker	(56)
				(57)
				(58)
	Umbilical cord blood	↓miR-210		(54)
		↓miR-374a		(59)
		↓miR-181b-5p ↓miR-374a-5p ↓miR-376c-3p		(60, 61)
		↓miR-181b		(60, 61)
		↑miR-210		
	Maternal whole blood			(55)
Pig	Blood plasma			(58)
Rat	Cerebral cortex		Inhibition of miR-210 as therapy	(62)
				(63)
				(64)
		↓miR-139-5p	miR-139-5p agomir as therapy	(65)
		↓miR-129-5p	miR-129-5p mimic as therapy	(66)
		↓miR-23a/b ↑lncRNA-GAS5	**↑**miR-23a by ↓GAS5 as therapy	(67)
		↓miR-17	IRE1α RNase inhibitor and miR-17-5p mimic as therapy	(68)
		↓miR-17-5p	PPAR-β/δ agonist GW0742 as therapy	(69)
		↑chr6:48820833|48857932	circRNAs as diagnostic tools and therapeutic targets	(70)
	Hippocampus and cortex	↑lncRNAs BC088414	Inhibition of BC088414 as therapy	(71)
	Hippocampus	↓miR124 ↓miR132 ↓miR134	Maternal tRESV supplementation diet as neuroprotective strategy	(72)
	Pineal gland	↑miR-325-3p	Inhibition of miR-325-3p as therapy	(73)
Mice	Cerebral cortex	↓miR-23a/b ↓miR-27a/b	Therapeutic targets	(74)
		↓miR-592-5p	miR-592-5p upregulation as neuroprotective strategy	(75)
	Hippocampus	↑Meg3	**↑**miR-129-5p silencing of Meg3 as therapy	(76)
*In vitro*	OGD-induced activated microglial cells	↓miR-21	Ectopic miR-21 as therapy	(77)

*HI, hypoxia-ischemia; miR(NA), micro RNA; PPAR-β/δ, Peroxisome proliferator-activated receptor beta or delta; Procr, endothelial protein C receptor; tRESV, trans resveratrol*.

#### LncRNAs

Long-non coding RNAs (LncRNAs) are ncRNAs larger than 200 nucleotides that act as a competing endogenous RNAs controlling gene expression by sponging miRNAs, and binding and inactivating chromosomes (78). LncRNAs fold into complex secondary and tertiary structures defining their interactions and function, providing a scaffold for proteins to form regulatory complexes (79, 80). Studies have demonstrated that LncRNAs play an important role in the regulation of gene expression, particularly during CNS development (81), with nearly 40% of LncRNAs reported to be specifically expressed in the CNS and involved in brain development and related disorders (82, 83).

### Histone Modifications

DNA is wrapped around octamers of histone (H) proteins containing two copies of H2A, H2B, H3, and H4 proteins forming nucleosomes that are bounded by H1 linker histones. H3 and H4 have N-terminal tails that extend beyond the nucleosome and are permissive to modifications, such as acetylation, methylation, glycosylation, ubiquitination, farnesylation, citrullination, and ADP-ribosylation. These modifications alter the charge of the amino acid residues of histones, resulting in relaxed DNA (euchromatin), accessible for the transcriptional machinery, or condensed DNA (heterochromatin) where the transcriptional machinery cannot access (84).

Altogether, these modifications encode the histone code, that, depending on the type and locus of the modifications, the relative location of histones within or toward a gene, and the combination of histone modifications, can lead to enormously diverse readouts (85). Histone modifications can act in different ways depending on the context, increasing the complexity of the histone code. The best-described histone marks in HI brain damage are histone acetylation and methylation.

#### Histone Methylation

Many enzymes participate in the control of histone methylation, including histone methyltransferases (writers), histone demethylases (erasers), and proteins that recognize the methylated state (readers) (86).

Histone demethylation is carried out by histone lysine demethylases (KDM2–7), which can remove both activating and repressing methyl groups from the chromatin (87, 88). Studies exposing cell lines to hypoxia have shown increased expression of the KDM3 gene, which removes methyl groups from repressive H3K9 sites and activates gene expression (89, 90). Methyl modifications take place mostly on lysine (K) residues of H3 and H4, being the main histone methylation site subject to epigenetic variation H4K20 (91). Interestingly, *in vitro* experiments suggest that JmjC enzymes can act as molecular oxygen sensors in the cell (92).

#### Histone Acetylation

Histone acetylation by histone acetyltransferases (HATs) relaxes chromatin formation, promoting transcription, whilst the deacetylation of histone tails by histone deacetylases (HDACs) causes the chromatin to condense, thereby inhibiting transcription (93). HATs exert their effects in collaboration with proteins like p300/CBP, PCAF, SRC that can associate and regulate the transcription of HIF-1α (94). HDACs are divided into 4 classes based on their domain organization. Class I, II, and IV HDACs depend on zinc as a co-factor, whilst class III HDACs known as sirtuins (SIRTs), depending on the co-factor NAD+ (95). Class I and class IIa HDACs enhances HIF-1α stability by directly binding to the oxygen-dependent degradation domain of HIF-1α and class IIb HDACs promote HIF-1α transcriptional activity (96, 97).

## Potential Biomarkers

### miRNAs

MIR210 is the master hypoxamir and its expression is induced under hypoxia in many cell types. MIR210 acts downstream of HIF-1α, repressing several key processes to lower cellular energy requirements during hypoxia (31, 47, 98, 99). Human studies have consistently shown MIR210 upregulation in placenta (57, 100–103) and plasma (104) from preeclampsia pregnancies, and also in intrauterine growth restriction (56) (Table 1). Studies using maternal miRNAs derived from the placenta circulate in the maternal blood during pregnancy and may serve as non-invasive biomarkers. Studies using maternal plasma (57), and blood (55) have shown an elevation in MIR210 in both chronic and acute fetal hypoxia. miRNAs produced in the placenta circulate in the maternal blood during pregnancy and can be used as non-invasive biomarkers for hypoxia *in-utero* and HIE, allowing early interventions.

When perinatal HIE is diagnosed, it is critical to grade the injury to decide whether or not to subject the patient to hypothermic treatment. The ideal biomarker for HIE will quickly define the grade of HIE, and it should discriminate newborns with mild HIE, for whom hypothermia therapy is not indicated, from newborns with moderate HIE, who are eligible for this treatment. Levels of MIR210 in neonatal patient blood in combination with MIR374a, S100B protein, and Neuron-specific enolase (NSE) have shown high accuracy in distinguishing HIE patients from healthy newborns, but also between mild, moderate, and severe HIE (54). These biomarkers also showed prognostic value as their levels correlated with neonatal behavioral neurological assessment scores. Increased levels of MiR210 were corroborated in plasma using piglet newborn model of HI (58).

Notably, MIR374a is downregulated in the umbilical cord blood after global HIE in humans (59). The extent of down-regulation of MIR374a corresponded to the severity of the insult and, as such, combining the levels of MIR374a with MIR210 may have prognostic value. Other studies in human neonates have shown a correlation between the downregulation of MIR181b, MIR199a, and MIR376c in the umbilical cord blood after HI, and the severity of the hypoxic insult (60, 61). Combining the levels of MIR181b and its target ubiquitin C-terminal hydrolase-L1 (*UCH-L1*) has diagnostic value, possibly enabling discrimination between moderate and severe HIE.

A principal component of HI brain damage is the inflammatory response and associated excitotoxicity (105). HI activates astrocytes and microglia that participate in the inflammatory response leading to increased levels of pro-inflammatory cytokines (106, 107). In this context, a second key hypoxamir is MIR21, a miRNA that could modulate inflammation. MIR-21 also controls several other key processes after HI, including cellular proliferation and migration, mitochondrial function, apoptosis, and HIF-1α stabilization and signaling (47, 49). MIR21 has been shown to be increased in cases of severe preterm fetal growth restriction compared to controls, and combined expression profiles of MIR21 and MIR20b in maternal blood were shown to be associated with the grade of fetal hypoxia at birth (55).

For the use of miRNAs as biomarkers, optimization of protocols for extraction and analysis of circulating miRNAs still needs further improvement toward selectivity and specificity (108). Remarkably, a study in human neonates demonstrated that it is feasible to extract sufficient miRNA from a single dried peripheral blood spot (109), with expression patterns that correlate well with those from EDTA-blood. This method eliminates potential sources of error, associated with blood collection and centrifugation. It is relatively cheap, technically easier to obtain, and easier for transportation and storing. Optimization and standardization of protocols for miRNA analysis will help the implementation into a clinical setting.

One consideration when examining miRNAs profiles after HI is that people of discrepant ethnicities might respond to the injury in different ways, therefore study results obtained in one ethnic group might not be appropriate for another cohort (54). Large-scale studies covering diverse ethnicities should be conducted to address this question.

### LncRNAs

In addition to ncRNAs, elevated levels of circulating cell-free fetal DNA (cffDNA) and cell-free fetal RNA (cffRNA) have been proposed as an early indicator of damages caused by perinatal hypoxia, that could be used as early biomarkers for preeclampsia or HIE. A study using newborn piglets exposed to hypoxia-reoxygenation revealed tendencies to higher concentrations of cffDNA in the cerebrospinal fluid in comparison to controls (110). This indicates that CffDNA and cffRNA levels in maternal blood, and also cell-free RNA of placental origin, could have potential applications as biomarkers for the screening and diagnosis of preeclampsia (111).

## Potential Treatments

### HIF-1α

In the brains of both fetuses and posnatal (P) 12 rat pups, fetal hypoxia resulted in global DNA hypomethylation and a continuous increase in HIF-1α mRNA and protein, and increased brain injury in response to hypoxia and ischemia (112). In the early stages of neonatal cerebral ischemia, inhibition of HIF-1α has been shown to be neuroprotective. In P7 HI rats inhibition of HIF-1α by 2-methoxyestradiol (2ME2) immediately after HI protected neuronal cells, attenuated blood-brain-barrier (BBB) disruption, and reduced brain edema (113). In contrast, the stabilization of HIF-1α with dimethyloxalylglycine (DMOG), increased BBB permeability and brain edema.

The c-glycosylated flavonoid, vitexin (5, 7, 4-trihydroxyflavone-8-glucoside) is a natural compound found in many medicinal plants, that has HIF-1α inhibitor activity (114). Intraperitoneal administration of vitexin immediately (5 min) after the HI insult in perinatal rats attenuated the increase in HIF-1α and vascular endothelial growth factor (VEGF), reduced infarct size, improved brain edema, BBB disruption, and neuronal cell death, and improved the neurobehavioral outcomes (115). Pretreatment with vitexin before HI showed the same results (116), diminishing the pro-apoptotic signaling pathway by inhibiting the phosphorylation of Ca^2+^/Calmodulin-dependent protein kinase II, and increasing the BCL-2/BAX protein ratio 24 h after injury. Animals pretreated with vitexin showed reduced brain infarct volume, brain atrophy, and improved neurobehavioral outcomes. Vitexin has also been proposed as a treatment for HI induced epilepsy (117). Treatment with vitexin suppress brain HI induced electrical activity in neonatal rats, by inhibiting the Na-K-Cl cotransporter (NKCC1), and preventing HI induced BBB leakage, and inflammatory cytokine and neutrophil infiltration. These results support further scientific exploration of vitexin as therapy for perinatal for HIE.

### DNA (de)Methylation

Defining the direct vs. indirect effects of hypoxia on DNA methylation in HIE populations is challenging. DNA methylation is time, tissue, and cell-type-specific, which poses a challenge in human studies that usually analyze peripheral blood (118). Studies in animal models have shown a causal effect of gestational and perinatal acute hypoxia on the regulation of gene-specific DNA methylation in mediating the neonatal programming of hypoxic sensitivity and the resulting consequences on the developing fetus and offspring. The first report associating DNA methylation to HI brain damage came from a model of ischemia/reperfusion using adult rats, showing that HI generated a 3- to 4-fold increase in methyl group incorporation in the brain (119). Transgenic animals expressing reduced DNA methyltransferase levels did not show this increase in DNA methylation and were resistant to HI brain injury.

In addition, evidence from studies following stroke indicated that the generation of reactive oxygen species (ROS) and reactive nitrogen species (RNS) directly modify cytosine residues chemically, by promoting DNA hydroxymethylation (120, 121). Moreover, peroxides involved in stroke have been shown to induce nucleobase modifications like 5-chlorocytosine, which mimics 5-mC and induces improper *Dnmt1* methylation within CpG sequences, resulting in gene silencing (122). These findings associated oxidative stress and epigenetic changes via chemical DNA modifications and altering DNA-protein interactions.

In the perinatal period, the majority of studies involving DNA methylation in HI have investigated the effect of a preconditioning stimulus on methylation and subsequent vulnerability to the HI insults. In ischemic tolerance, exposure to a sublethal ischemic (preconditioning) event protects the brain against a subsequent severe ischemic challenge, producing tolerance.

In rat studies, mild fetal asphyxia during the last week of gestation caused global changes in gene transcription at birth, with down-regulation of most mRNA transcripts in the brain, and upregulation of DNMT1 and DMT3L, various HDACs, the Polycomb group ring finger 2 (PCGF2) and the methyl-CpG-binding protein-2 (MeCP2) (123). Fetal asphyxia preconditioning protected against subsequent severe perinatal global HI, reducing postnatal mortality and behavioral deficits after perinatal HI. Concomitantly, fetal asphyctic preconditioning lowered acute cytokine infiltration and modulated the transcriptional response to perinatal asphyxia. This effect was mediated by epigenetic changes, particularly involving histone deacetylation (124–126).

Other evidence suggest that prenatal insults generally tend to increase vulnerability to neonatal HI. In studies using rat models, HI induced gene-specific DNA hypermethylation. In the developing rat fetus, fetal hypoxia (GD 15 to 21) increased methylation of the glucocorticoid receptor (GR) gene (*Nr3c1*) promoter and repression of the GR in the brain, leading to an increased brain vulnerability to hypoxic-ischemic injury (127). In this way, methylation controls the expression patterns of GR, being key in the stress-mediated programming of GR expression (127, 128).

As mentioned, HIF-1α and DNA hypomethylation participate in fetal stress-mediated programming of HI sensitive phenotypes (112). Maternal hypoxia in rats, during the last week of gestation, reduced global methylation levels in the fetal brain and affected methylation of the HIF-1α gene. More specifically, hypomethylation induced by either maternal hypoxia, or pharmacological treatment with the DNMT inhibitor 5-aza-dC increased the vulnerability of the fetus to subsequent neonatal HI and worsened neurobehavioral outcomes in the rat pups. Interestingly, inhibiting HIF-1α with 2ME could counteract some of the damaging effects of hypomethylation.

Prenatal nicotine exposure has shown to increase brain infarct size after subsequent neonatal HI in male rats (129, 130). This increased susceptibility was linked to a down-regulation of angiotensin II receptor expression in the brain and hypermethylation of the angiotensin II type 2-receptor (*At2r*) promoter.

### miRNAs

miRNAs and its regulated genes have also been proposed as therapeutic targets. Induction of miRNAs inhibiting pro-apoptotic pathways and inhibition of miRNAs implicated in HI-induced inflammation and apoptosis has shown therapeutic potential in animal models. Further characterization of these findings will help them advance into clinical trials.

MiR139-5p is down-regulated in P10 rat brains after HI treatment, and in cultured neurons exposed to OGD (65). The expression of MiR139-5p correlates inversely with the expression of one of its targets, the pro-apoptotic protein human growth transformation dependent protein (HGTD-P) (131). Interestingly, administration of MiR139-5p agomir attenuates HI brain damage, which is concurrent with the downregulation of HGTD-P expression. This effect was shown even at 12 h after the insult, indicating that targeting epigenetic pathways could extend the therapeutic window in HI brain damage.

The selective α2-adrenoreceptor agonist dexmedetomidine has been shown to provide neuroprotection in HI by inhibiting apoptosis, oxidative activity, Notch/NF-κB activation (132), and inflammation (133). MiR129-5p targets the type III procollagen gene (*COL3A1*) and has shown therapeutic potential in P7 HI rats mimicking and enhancing the neuroprotective effect of dexmedetomidine (66).

Hypoxia-induced brain injury appears to downregulate the expression of the MiR23-27 cluster leading to increased apoptosis. Overexpression of MiR23a/b and MiR27a/b was shown to exert neuroprotective effects in the late stage of mouse gestation (GD20) after global maternal hypoxia, by reducing apoptotic pathways including the expression of Apoptotic protease factor-1 (Apaf-1) (74). Indirectly increasing MiR23a expression by reducing the expression of the lncRNA Growth arrest-specific 5 (GAS5), that binds to MiR23a, reduced infarct size after HI in P10 rats (67).

One pathological mechanism of HI damage is the induction of endoplasmic reticulum stress, which activates the unfolded protein response (UPR). The UPR induces activation of stress sensor signaling pathways, like the RNase inositol requiring enzyme-1 alpha (IRE1α) pathway, leading to inflammation and neuronal cell death. In this context, MiR17-5p, a substrate of IRE1α, is a target that has shown therapeutic potential (68). Animal studies have shown that MiR17-5p mimic before HI, prevented inflammasome activation reducing brain infarct volume. Moreover, intranasal administration of the IRE1α inhibitor STF-083010 1h post-HI attenuated MiR17-5p downregulation and brain injury, and improved neurological behavior outcomes. It has also been reported that activation of the nuclear receptor peroxisome proliferator-activated receptor beta/delta (PPAR-β/δ) in P10 HI rat, by intranasal delivery of the agonist GW0742, could induce MiR17-5p levels (69), diminishing apoptosis, brain infarct area, brain atrophy, and improving neurological function post HI.

*In vitro* findings showed that primary neonatal rat microglial cells exposed to hypoxia experience downregulation of MiR21 expression and upregulation of one of its targets, the apoptosis-inducing factor Fas ligand (*FasL*) (77). Overexpression of FasL post hypoxic microglial activation increased neuronal apoptosis, which can be partially reversed by ectopic expression of MiR21. MiR592-5p targets inflammation by targeting the prostaglandin D2 receptor. HI in P7 rats reduces the expression of MiR592-5p and increases prostaglandin D2 expression in the hippocampus, which is detrimental (75), induction of this miRNA could also be beneficial in HI.

MiR124 is the most abundant brain-specific miRNA and plays key roles in neuronal development. Multiple studies have shown a neuroprotective effect of MiR124 in adult HI models (134–137), which has not been validated for the perinatal period. Animal studies showed that MiR124 was down-regulated in the hippocampus after global neonatal hypoxia in rats (72). MiR124 warrants further investigation in neonatal HI models as it may have therapeutic value.

A potential strategy to use miRNAs as therapeutic targets is comprised of inhibiting miRNAs by using antagomirs, or naturally occurring compounds with similar effects such as curcumin or resveratrol. MiR210 expression steadily increases over the first 24 h after HI in P10 rats, disrupting glucocorticoid receptor-mediated neuroprotection and increasing leakiness of the BBB (62–64). Inhibition of MiR210 using an intracerebroventricular injection of complementary locked nucleic acid oligonucleotides increased BDNF signaling, reduced neuronal death and infarct size, and improved functional recovery of the animals. *In vitro* studies also support these findings (138). Although this suggests that MiR210 inhibitors could be used as a potential therapy in HI brain injury, further characterization of its spatiotemporal expression, regulation, targets, and physiological and pathogenic effects in HIE is still required.

NF-κB triggers MiR155, inducing pro-inflammatory effects compromising the BBB integrity. Inhibition of MiR155 is neuroprotective in adult HI models by reducing brain cytokines (139), but its role in neonatal models is underexplored to date. The neuroprotective actions of resveratrol and curcumin in neonatal HI (140–142) may in part be due to the downregulation of MiR155 and MiR21 by resveratrol, and the downregulation of MiR21 by curcumin, combined with epigenetic and anti-oxidant mechanisms (143, 144). MiR153 is reported to be a neuron-related miRNA (50). Inhibition of MiR153 protects neurons against OGD/R-induced injury by increasing the expression of Nuclear factor erythroid 2-related factor 2 (*Nrf2*) and heme oxygenase-1 (*HO-1*) signaling.

Sleep problems associated with circadian rhythm disruptions are common in children after mild-moderate HIE (145), and disruptions in miRNAs likely contribute to these abnormalities. HI in P7 rats leads to an increased expression of MiR325-3p in the pineal gland (73). This disrupts melatonin signaling by targeting Aralkylamine N-acetyltransferase (AANAT), a key protein that controls melatonin synthesis, and this leads to circadian rhythm disturbances. Reducing MiR325-3p has been shown to prevent this impairment after OGD *in vitro*.

### Long-Non Coding and Circular RNAs

Studies in human neonates diagnosed with HIE and animal models have shown that lncRNAs are aberrantly expressed under hypoxic conditions and might be implicated in regulating the expression of protein-coding genes involved in pathological processes associated to HI brain damage. A microarray study in human neonates showed that neonatal HI dramatically changed the expression patterns of numerous ncRNAs in peripheral whole blood (146), including 376 lncRNAs and 126 mRNAs involved in the immune system and nervous system. A study on the P7 HI rat cortex identified 7,157 differentially expressed mRNA transcripts, and 328 differentially expressed lncRNAs targeting mRNAs involved in inflammatory and immune responses, wounding, and neurological system processes (147). Specifically, JAK-STAT, NF-κB, and TLR signaling pathways were altered. Targeting genes in these pathways is proposed as a therapeutic strategy.

LncRNAs have shown potential therapeutic applications in animal models. Various studies have pointed to the inhibition of HI-induced lncRNAs as a therapeutic approach to induce neuroprotection. A second microarray profiling study in P10 rats exposed to HI pointed in the same direction, finding expression differences in 322 lncRNAs and 375 coding genes in hippocampal and cortex tissue 24 h after the insult (71). Upregulated protein-coding genes were also involved in inflammation and wounding, while repair and neurogenesis pathways were downregulated. BC088414, which plays a role in apoptosis by regulating caspase 6 (*Casp6*) and in adrenergic signaling by regulating the beta-2 adrenergic receptor (*Adrb2*), was the most significantly upregulated lncRNA. Inhibition of BC088414 with a siRNA in PC12 cells exposed to OGD resulted in the downregulation of *Adrb2* and *Casp6*, increased cell proliferation, and decreased apoptosis.

The lncRNA maternally expressed gene 3 (Meg3) induces cell death in ischemia by binding to the p53 DNA binding domain (148). A study in P7 HI mice hippocampus showed that the Meg3 sponges MiR129-5p, a miRNA with neuroprotective activity, and abolishes the effect of dexmedetomidine therapy (76). The silencing of Meg3 and upregulation of MiR129-5p enhanced the therapeutic effect of dexmedetomidine. Finally, the lncRNA Growth arrest-specific 5 (Gas5) was also shown upregulated in HI models (67). Gas5 sponges MiR23a thereby preventing its neuroprotective action. Inhibition of Gas5 by intracerebroventricular delivery of Gas5 small hairpin RNA has been shown to reduce brain infarct size and diminish functional sequelae in rats, suggesting its use as therapy for the treatment of HI brain injury.

CircRNAs are a subtype of lncRNAs that form a closed loop. CircRNAs play crucial roles as miRNA sponges, and recent studies highlighted the role of these molecules in hypoxic regulation. An exploratory study in P3 HI rats found 98 dysregulated circRNAs in the brain (70). One of the top hits, termed chr6:48820833|48857932, targets HIF-1α signaling by sponging the HIF-1α-targeting-miRNAs MiR433-3p and MiR206-3p. Another study in P10 HI rats discovered a total of 66 circRNAs differentially expressed in HI brain damage rats compared to controls (149). Numerous mRNAs transcribed from the host genes of altered circRNAs were associated with brain damage and neural regeneration processes. These results indicate a novel focus for future studies investigating the molecular mechanism underlying HIE and potentials new biomarkers and treatments through modulating circRNAs.

### Histone Methylation

Enhancer of zeste homolog 2 (EZH2), a catalytic subunit of Polycomb repressive complex 2 (PRC2), plays an important role in mammalian CNS development (150). EZH2 participates in hippocampal learning, memory, and neurogenesis through trimethylation at H3K27 (H3K27me3), which silences downstream genes, including, among others, BDNF and PTEN (151). In HIE, autophagy is increased in the hippocampus, and inhibition of autophagy provides neuroprotection. In this regard, volatile anesthetics, i.e., sevoflurane and isoflurane, have shown to be neuroprotective against HI brain damage in neonatal rats (152, 153) (Table 2). Diverse studies have shown sevoflurane inhibits excessive hippocampal autophagy increasing the expression of EZH2, H3K27me3, and decreased expression of PTEN induced by HI, improving the behavioral outcome (154).

**Table 2:** Clinical implications of histone modifications in perinatal hypoxia ischemia studies.

**Observed effect in HI**	**Observed in**	**Other findings**	**Clinical implications**	**References**
↓EZH2 ↑H3K27 3me	Rat hippocampus	↑ Pten/Akt/mTOR pathway **↑** Autophagy	Sevofluorane neuroprotection via **↑** EZH2	(152–154)
↓SIRT1	Rat hippocampus and cerebral cortex	**↑** Unfolded protein response (UPR)	Melatonin neuroprotection via **↑** SIRT1	(155)
↑ SIRT1	Mice oligodendrocyte progenitor cells	↑ Cdk2/Rb/E2F1 pathway ↑ Oligodendrocyte progenitor cell proliferation	↑ Sirt1 activity for oligodendrocyte recovery	
↑Acetyl-Histone H3 (Ac-H3) ↑Acetyl-Histone H4 (Ac-H4)	Rat	↑Caspase-3 ↑Apopstosis	HDAC activity as target of uridine neuroprotection	(156)
↑Histone 3 deimination (citH3)	Mice hippocampus, cortex, striatum and piriform cortex	↑ TNFα	PAD inhibition as therapeutic target	(157, 158)
↑REST	Hippocampal CA1	↓GluR2 ↓AMPA and NMDA receptors ↑Excitotoxicity	REST inhibition as therapeutic target	(159, 160)

*SYMBOLS: ↑, Upregulation; ↓, Downregulation; EZH2, Enhancer of zeste homolog 2; HI, hypoxia-ischemia; HDAC, Histone deacetylase; PAD, protein arginine deiminases; REST, RE1-Silencing Transcription factor; SIRT1, Sirtuin 1; TNFa, Tumor necrosis factor alpha*.

### Histone Acetylation

Many studies have shown the implications of HATs in perinatal HI damage. Fetal asphyxia in E17 rats leads to an upregulation of class I HDACs HDAC1, HDAC2, HDAC3, and the HAT MYST3 (123). The same stimulus upregulated HDAC1 and class IIb HDAC10 and HDAC11, after severe perinatal asphyxia (126). The HDAC SIRT1 plays an important role in the regulation of oligodendrocyte progenitor cell proliferation and oligodendrocyte regeneration after neonatal brain injury. Neonatal hypoxia in P3 mice has been shown to enhance SIRT1 and SIRT1/Cdk2 complex formation through HIF1α activation, leading to an enhanced oligodendrocyte progenitor cell proliferation (161). Enhancing SIRT1 activity may promote oligodendrocyte recovery after diffuse white matter injury. SIRT1 expression was significantly reduced after neonatal HI in P7 rat pups (155) in cells that activate the unfolded protein response (UPR). In that same study, melatonin neuroprotection involved the prevention of SIRT1 downregulation and UPR activation.

From a therapeutic perspective, HDAC inhibitors (HDACi's) represent the most widely studied epigenetic drugs as several existing marketed drugs and natural dietary metabolites inhibit HDACs. Regarding HI, HDAC inhibition reduces HIF-1α signaling, which is generally protective against HI (96, 112). Numerous HDAC inhibitors have shown beneficial effects in either focal or global neonatal HI. For example, the marketed anticonvulsant sodium valproate inhibits class I HDAC activity and has shown potential in treating neonatal HI in rats (162). Several naturally occurring bioactive molecules, such as trichostatin A (163), sodium butyrate (164), curcumin (142), quercetin (165), resveratrol (72, 140, 141), and uridine (156) have also proven protective effects in focal neonatal HI, the effects of which were shown to be at least partially dependent on HDAC inhibition. The mechanism(s) by which HDACi's provide neuroprotection include prevention of oxidation, suppression of inflammation, and reduction of apoptosis. Importantly, histone acetylation/methylation precedes DNMT or TET methylcytosine dioxygenase binding and promoter methylation/demethylation, and these mechanisms function cooperatively (166). Still, many potential problems remain to be addressed before clinical use of selective HDACi's for the treatment of HIE.

### Other Histone Modifications and Epigenetic Complexes

Protein deimination (citrullination) is a post-translational modification that converts the amino acid arginine into citrulline, is caused by Ca^+2^-regulated peptidylarginine deiminases (PADs), and can act on histone tails.

H3 citrullination (citH3) by PAD4 is associated with gene regulation and the formation of neutrophil extracellular traps in response to infection. PAD activity is induced under HI with or without lipopolysaccharide stimulation. Selective and targeted pharmacological PAD inhibition following HI can be a therapeutic target to enhance neuroprotection (157). Hypothermia following the insult inhibits this pathway and affect the Ca^2+^-regulated PAD activation (158).

A large degree of epigenetic variation is controlled by epigenetic regulatory complexes. Several epigenetic regulatory complexes may play a role in neonatal HI injuries. The repressor element-1 (RE1) silencing transcription factor (REST) is the main regulator of neurogenesis and neuronal fate. Neuronal genes controlled by REST contain an RE1 motif. As such, REST assembles to this site with its co-repressor CoREST and recruits a number of epigenetic regulators including HDACs and histone methyltransferases to the promoters of target genes to achieve epigenetic changes (167, 168). It has been demonstrated that REST is a HI-responsive gene, regulating around 20% of the hypoxia-repressed genes. As an example, HI has been shown to upregulate REST expression in the hippocampal CA1, suppressing GluR2 gene expression (159). Interestingly, the downregulation of REST expression with antisense oligodeoxynucleotides has been demonstrated to be neuroprotective 72 h post OGD. REST was also shown to orchestrate epigenetic changes and silencing of miR-132 in insulted CA1 neurons (169) and has been shown to bind directly to the HIF-1α promoter in order to repress HIF-1α transcription after prolonged hypoxia (159, 170, 171). Moreover, REST has been demonstrated to repress the transcription of AMPA and NMDA glutamate receptor subunits (168), which are involved in mediating excitotoxicity after HIE (18). Recently, it has been shown that adult rats overexpress REST after CAO (160), and knocking down REST with an intracerebral siRNA injection increased the expression of its target genes, attenuated apoptosis, and infarct volume, and improved post-ischemic functional recovery. Altogether, although the actual therapeutic potential of REST in the perinatal period has not yet been explored, these data suggest REST reflects a promising new therapeutic target to treat acute hypoxic brain damage.

## Discussion

There is an urgent need for new therapies for neonatal HI, as currently, therapeutic hypothermia for term infants is not fully protective against HIE, and the disability burden after neonatal HI remains high. Evidently, epigenetic mechanisms play a key role in the pathological cascade after neonatal HI. The cellular hypoxia response, mediated by HIF-1α and other hypoxia-inducible factors, is controlled by an intricate multi-leveled epigenetic network centered on HIF-1a. A better understanding of this intricate regulatory circuit will provide us with new diagnostic tools and therapeutic approaches for HIE. As an example, HIF-1α signaling controls the expression of various miRNAs, whileHIF-1α by itself is intricately controlled by different miRNAs. Apart from HIF-1α signaling, numerous miRNAs regulate a myriad of other processes relevant for HI, ranging from well-studied processes like inflammation and apoptosis to circadian rhythm disturbances after HIE. Some hypoxia-regulated miRNAs in the maternal blood are promising in terms of identifying pregnancies at risk of fetal hypoxia, permitting early intervention, whereas preclinical interventions using miRNAs are promising in combination with therapeutic hypothermia. Together with miRNAs, HDACs hold promise as biomarkers and therapeutic targets. HDAC inhibitors represent the most advanced agents in this respect as several existing marketed drugs and natural dietary metabolites have been shown to directly inhibit HDACs.

Still, no single epigenetic mark has demonstrated enough reliability and reproducibility to be used as a biomarker or as a therapeutic target in a clinical setting. For this purpose, replication and/or validation studies in larger cohorts are needed. In conclusion, even though numerous advancements have been made in understanding the pathophysiology of perinatal HI brain damage, still our knowledge on the role of epigenetics in HI is very limited. As such, new discoveries on epigenetics may mark the beginning of an etiopathogenic research revolution in neurodevelopmental disorders, and continued exploration of this area is of great promise.

## Author Contributions

MBu, MBa, MAB, AG, CL, and DH conceived the presented idea. MBu and MBa wrote the first draft. DH and HS edited the draft. AG, MAB, and CL provided funding for the manuscript. All authors read the final version of the manuscript.

## Conflict of Interest

The authors declare that the research was conducted in the absence of any commercial or financial relationships that could be construed as a potential conflict of interest.

## Abbreviations

BBB: blood-brain barrier
DNMTs: DNA methyltransferases
E: embryonic day
HDACs: histone deacetylases
HI: hypoxia-ischemia
HIE: hypoxic-ischemic encephalopathy
HIF: hypoxia inducible factor
HIF-1α: Hypoxia-inducible factor 1α
HRE: hypoxia response element
JmjC: Jumonji-C histone demethylase
KDMs: histone lysine demethylases
P: postnatal day
REST: repressor element-1 (RE1) silencing transcription factor
TETs: ten-eleven translocation enzymes
5-aza-dC: 5-aza-2′-deoxycytidine.
